# Validity and Reproducibility of the Sargent Jump Test in the Assessment of Explosive Strength in Soccer Players

**DOI:** 10.2478/v10078-012-0050-4

**Published:** 2012-07-04

**Authors:** Paulo Gil da Costa Mendes de Salles, Fabrício Vieira do Amaral Vasconcellos, Gil Fernando da Costa Mendes de Salles, Renato Tavares Fonseca, Estélio Henrique Martin Dantas

**Affiliations:** 1 Universidade do Rio de Janeiro (UNIRIO) – Laboratório de Biociências da Motricidade Humana (LABIMH) - Brazil.; 2 Universidade Federal do Rio de Janeiro (UFRJ) – Faculdade de Medicina – Rio de Janeiro, Brazil.

**Keywords:** soccer, validity, reproducibility of the results, muscular strength

## Abstract

The purpose of this study was to check the validity and the intra- and inter-evaluators reproducibility of the Sargent Jump Test, as an instrument of explosive strength measurement of soccer players of the sub-15 class. Forty-five soccer players were randomly selected from different clubs competing in the local soccer championship. All subjects performed one test on the same jump platform model Jumptest^®^ (Hidrofit Ltda, Brazil) and two independent Sargent Jump Tests assessed by the same evaluator. Two days later, another Sargent Jump Test was performed simultaneously assessed by 2 evaluators. In all tests, three jumps were performed and the highest one was registered. In order to check the validity, the first Sargent Jump Test results were compared to those from the jump platform, considered the gold standard. To evaluate intra- and inter-evaluator reproducibility, results from the first, second and third Sargent Jump Tests were analyzed. The validity and reproducibility were evaluated by intraclass correlation coefficients (ICC), and by the Bland and Altman test (statistical pack SPSS 11.0), with a significance level set at p<0.05. The values found for validity (r=0.99, p=0.001), for intra-evaluator reproducibility (r=0.99, p=0.001) and for inter-evaluator reproducibility (r=1.0, p=0.001), permitted us to conclude that the Sargent Jump Test is a valid and reproducible instrument for measuring the explosive strength in homogeneous groups, such as those used in the present study.

## Introduction

Explosive strength is an ability which varies according to the ratio between movement velocity and the developed strength by the specific muscle groups (Newton and Kraemer, 1994; Stone et al., 2003). Regarding soccer, explosive strength is of great relevance to physical capacity, as the athlete often needs to perform actions as quickly as possible, with high intensity (Gissis et al., 2006;).

During the decades of the 60’s and 70’s, several investigators (Cavagna et al., 1965; 1968; Cavagna, 1970; Melvill and Watt, 1971; Asmussen and Bonde-Petersen, 1974; Asmussen, 1974; Ford et al., 1976) established procedures in which the vertical jump was used as a method for evaluating explosive strength. Wisloff et al. (2004) showed a strong relationship between the vertical jump, which focuses on explosive strength of the lower limbs, and sprints in competitive soccer players.

According to Malliou et al. (2003), the vertical jump test can be performed either in a laboratory, with the use of jump platforms (JP), or in the field, using the Sargent Jump Test (SJT), in order to measure explosive strength of the lower limbs of athletes from a variety of sports (Socha et al., 2006). However, the validity of the SJT in relation to the gold standard JP test in young soccer players, as well as the intra- and inter-evaluator reproducibility on the SJT has never been examined. Hence, the purpose of this study was to evaluate the validity, intra- and inter-evaluators reproducibility of the SJT, as well as to assess explosive strength of the lower limbs in soccer players of the sub-15 class.

## Material and Methods

### Participants

This cross-sectional study included 45 soccer players from several clubs from the sub-15 soccer class in Rio de Janeiro, Brazil, randomly selected among approximately 500 athletes who comprised this class. The subjects aged 14.3 ± 0.66 years old, body mass of 59.5 ± 6.5 kg and body height equal to 168.4 ± 7.4 cm), agreed to participate in the study, and had to fulfill the following criteria: a) to be affiliated to the Soccer Federation of Rio de Janeiro for at least 1 year, b) to be participating in the soccer championship in Rio de Janeiro, and c) not to present any influential problems in obtaining and interpreting the data. After having been thoroughly informed about the experimental protocol, the parents (or guardians) of the athletes signed an informed consent, according to the Resolution 196/96 of the National Council of Health about research involving humans. The study was approved by the local institutional ethics committee under the number 0174/2008.

### Measures and Procedures

As preliminary procedures, body height and body mass of the volunteers were measured in order to assess sample homogeneity. Subjects were then randomly selected for the sequence of test performance. This sequence was maintained in the following tests. After receiving instructions about the test protocol, the volunteers participated in a 10-minute warm-up, which included moderate intensity running and jumping exercises of low intensity. Subjects performed four jump tests: one JP (JP1) and three SJTs. The first and second SJTs (SJT1 and SJT2) were performed on the same day as JP1, separated by an interval of at least two hours, based on suggestions by Skurvydas et al. (1999). These tests were assessed by the same investigator (A). The third SJT (SJT3) was performed two days after the previous tests, and was simultaneously assessed by two investigators (A and B), each one blinded to the results of the other. In all tests, three jumps were performed, and the highest one was used for further analysis.

According to the protocol described by Harman et al. (1991), in the JP, the subject was positioned with two feet on the platform, followed by a vertical jump, with free movements of the upper limbs and total freedom in joint flexion of the lower limbs. This strategic procedure was justified by the theoretical possibility that the subject could perform a higher jump, due to a greater impulse, than the biomechanical leverage would normally permit. All volunteers jumped three times, with a minimum interval of 45 seconds between the jumps, and the highest value was considered. To measure jump height, the JP Jumptest^®^ model (Hidrofit Ltda., Brazil) was used, as validated by Ferreira et al. (2008). The system of this platform determined the jump height via the flight time of the athlete, using the following equation: *height = 1/8 gt^2^*, where *g* is the gravity acceleration (9.81m/s^2^); and *t* is the actual time in the air (s). Photoelectric receptors distributed evenly on the inner area of the jump platform, counted the flight time from the moment the athlete left contact with the ground, when the light beam activated the receptors, to the time the subject’s feet landed on the platform and deactivated the light beam.

In order to assess the SJT performance, according to the protocol of Harman et al. (1991), the volunteers had their fingers on the right hand marked with orange chalk. While standing flat-footed next to a wall on their right side, and right arm extended above the head, the volunteer would mark on the wall the highest point that could be reached. At the moment preceding the jump, the volunteers could freely flex the lower limbs, as well as preparing the upper limbs for a sudden upward thrust, in effort to promote the highest vertical jump possible. At the highest point of the jump, the volunteers should extend the right hand against the wall as to mark the maximum height jumped. The jump height was the difference between the two points marked on the wall. All of the volunteers jumped three times, with a minimum interval of 45 seconds between the jumps and only the highest jump was considered. The same protocol was followed in all SJTs.

### Data analysis

Continuous data was described as means and standard deviations (SD) when normally distributed. To assess the normality of data distribution, the Kolmogorov-Smirnov test was used. The validity and the intra- and inter-evaluator reproducibility were evaluated by the intra-class correlation coefficient (ICC) and the Bland-Altman graphic method (Bland and Altman, 1986). The statistical package SPSS version 13.0 (SPSS Inc. USA) was used, with the significance level set at p<0.05.

The correlations between the results of the JP1 and SJT1 jumps were used to assess the validity of the SJT. The intra-observer reproducibility of the SJT was evaluated by correlating the SJT1 and SJT2 results; and for examing the inter-evaluator reproducibility of the SJT the results of each independent evaluator in the SJT3 were used.

## Results

Table 1 outlines the descriptive analysis of the anthropometric data of the 45 subjects studied. As can be seen from the Kolmogorov-Smirnov test, anthropometric data is normally distributed. Table 2 shows the descriptive analysis of all the jump test results.

**Figure 1:**
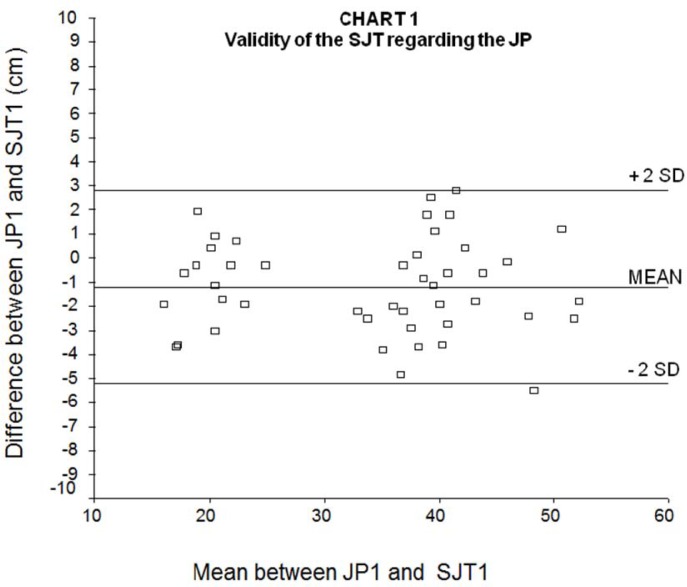
Bland-Altman graph of the validity of the Sargent jump test in relation to the Jump Platform test

The ICC between JP1 and SJT1, which reflects the validity of the SJT in relation to the JP test, was 0.99 (95% confidence interval: 0.97 – 1.00, p=0.001). Figure 1 shows the Bland-Altman graph between JP1 and SJT1. The mean difference between JP1 and SJT1 was −1.2 cm (SD: 2.0 cm), which means that 95% of the differences between JP1 and SJT1 lied between +3 and −5 cm (± 2 SD). The mean relative error, calculated from the ratio between the jump differences and the mean jumps (JP1 and SJT1) was 6%.

**Figure 2:**
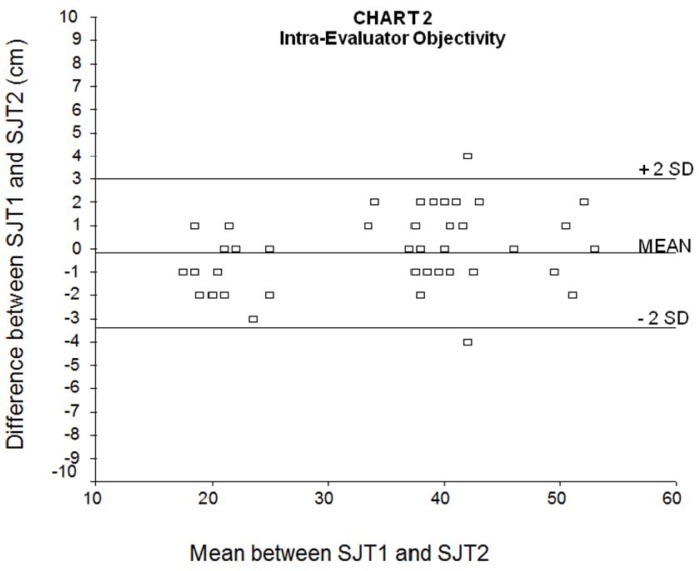
Bland-Altman graph of the intra-evaluator reproducibility of the Sargent jump test

The ICC between SJT1 and SJT2, which reflects intra-evaluator reproducibility of the test, was 0.99 (95% confidence interval: 0.99 – 1.00, p=0.001). Figure 2 shows the Bland-Altman graph between SJT1 and SJT2. The mean difference between SJT1 and SJT2 was −0.2 cm (SD: 1.6 cm), which means that 95% of the differences between SJT1 and SJT2 lied between +3 and −3 cm (± 2 SD). The mean relative error, calculated from the ratio between the jump differences and the mean jumps (SJT1 and SJT2) was 4%.

**Figure 3:**
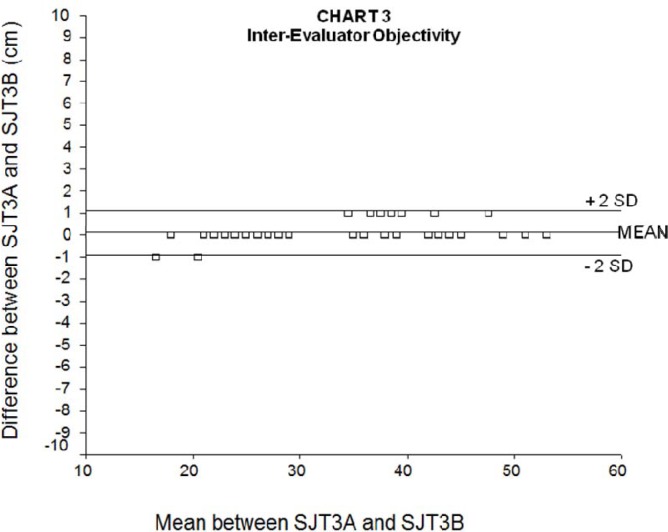
Bland-Altman graph of the inter-evaluator reproducibility of the Sargent jump test

The ICC between SJT3 results by different independent evaluators, which reflects inter-evaluator reproducibility of the test, was 1.00 (95% confidence interval: 0.99 – 1.00, p=0.001). Figure 3 shows the Bland-Altman graph between evaluator A and B on SJT3. The mean difference between evaluators was 0.1 cm (SD: 0.5 cm), which means that 95% of the differences between evaluator A and B lied between +1 and −1 cm (± 2 SD). The mean relative error, calculated from the ratio between the jump differences and the mean jumps (SJT1 and SJT2) was 0.7%.

## Discussion

The validation process of a measurement instrument should meet many criteria. Among them, we can consider that the reproducibility may be one of the most important and, thus, several authors have been testing this characteristic by similar protocols (Peter, 1979; Aziz et al., 2008). In studies regarding reproducibility, the greatest merit of the methodological approach developed seems to be the highest achieved correlation and reproducibility of the data, both for the same evaluator and between different evaluators. In the present study, the intra- and inter-evaluators reproducibility was assessed for measuring explosive strength of the lower limbs in young soccer players.

According to Bland and Altman (1986), it is expected that 95% of the differences among the jumps are between +2 SD and −2 SD. Hence, the size of the SD will influence the accuracy of the test, and the acceptable maximum size of differences among the jumps depends on the investigator’s judgment.

Thereby, when the SJT validity is analyzed in relation to the JP test, considered the gold standard power evaluation, there is a 95% chance that the SJT will present results between 5.2 cm higher to 2.8 cm lower than the results in the JP, and that the average SJT was 1.2 cm higher than the JP jump, representing a mean variation of 6%. We judge that this range of variation is very narrow, and therefore the SJT can be considered a valid alternative to the platform jump test to evaluate explosive strength of the lower limbs in our study population. This is confirmed by the very high intra-class coefficient of correlation found between the two methods.

Similarly, there is a 95% chance that, when assessed by the same person, one of the SJTs will be between 3.0 cm higher to 3.4 cm lower than the second SJT, and that when only one test is assessed by different evaluators, the result obtained by the second evaluator will be between 1.1 cm higher to 0.9 cm lower than the result obtained by the first evaluator. These results ascertain that the SJT is also highly reproducible both between different measures with the same evaluator and between different evaluators. This finding is confirmed by the very high intra-class correlation coefficients found between SJT1 and SJT2 results (same evaluator) and between different evaluators in SJT3.

The results of this study showed that, for soccer athletes of the sub-15 class, the SJT is as valid as a field test in assessing explosive strength of the lower limbs. Moreover, results showed there were no significant differences with intra- and inter-evaluators, which implies a high correlation and reproducibility among the results of these tests when performed by either a single evaluator or different evaluators. Taking into consideration the importance of explosive strength assessment of soccer players, the SJT can be considered a very useful and valid method for assessing power and jumping ability, especially due to its accessibility and feasibility.

## Figures and Tables

**Table 1:** Descriptive analysis of anthropometric data

	Body mass (kg)	Body Height (cm)
Mean	59.5	168.4
Standard Deviation	6.5	7.4
Variation Coefficient	10.9%	4.4%
Kolmogorov- Smirnov (p-value)	0.95	0.89

**Table 2:** Descriptive analysis of jump tests results (cm)

	Evaluator	Mean	SD	Min.	Max.	VC
JP1	A	33.36	10.86	15.1	51.2	32.5%
SJT1	A	34.53	10.92	17	53	31.6%
SJT2	A	34.69	10.45	18	53	30.1%
SJT3	A	35.09	9.87	16	53	28.1%
SJT3	B	34.96	9.70	17	53	27.8%

*Abbreviations: SD, standard deviation; Min, minimum value; Max, maximum value, VC, variation coefficient; JP1, jump platform test 1; SJT1, Sargent jump test 1; SJT2, Sargent jump test 2; SJT3, Sargent jump test 3*.
